# Services as a Determinant of Botswana’s Economic Sustainability

**DOI:** 10.3390/ijerph192215401

**Published:** 2022-11-21

**Authors:** Joseph Phiri, Karel Malec, Aubrey Sakala, Seth Nana Kwame Appiah-Kubi, Pavel Činčera, Mansoor Maitah, Zdeňka Gebeltová, Cathy-Austin Otekhile

**Affiliations:** 1 Department of Economics, Faculty of Economics and Management, Czech University of Life Sciences, 16500 Prague, Czech Republic; 2 Department of Economics, School of Humanities and Social Sciences, Copperbelt University, Jambo Drive, Riverside, Kitwe P.O. Box 21692, Zambia; 3 BEZK, z.s. Letohradská 669/17170 00 Praha 7, 17000 Prague, Czech Republic; 4 Department of Management and Marketing, Faculty of Economics and Management, Tomas Bata University, 76001 Zlin, Czech Republic

**Keywords:** services, economic development, GDP, ARDL Bounds test, Wald test, Botswana

## Abstract

In 2015, the services sector contributed about 58 percent to the gross domestic product (GDP) in Sub-Saharan Africa (SSA), which was a significant increase from the 47.6 percent observed in 2005, and a shift from the mining, agriculture, and manufacturing sector. This increase calls to support services as the catalyst for sustained economic development as indicated by the structural transformation and modernization theories. The main objective of this paper was to examine the relationship between and the impact of services on the economic development in Botswana and make recommendations on how Botswana can apply well-directed policies to improve its services sector and diversify its impact on other sectors and GDP, making it less reliant on mining which is vulnerable to price volatilities. The paper applied econometric modeling and results of the Autoregressive-Distributed Lag (ARDL) Bounds test for cointegration indicate that services and other industries services, agriculture, industry, mining, and investment impact GDP over the short and long run. These variables impacted GDP and converged to equilibrium at the speed of 46.89 percent, with a percent change in services in the short and long run impacting GDP by 0.328 and 0.241 percentages, respectively, and the outcome of the Wald test indicated causality from services to GDP growth. The services sectors have contributed over 40 percent to the country’s GDP from 1995 to the present, though the sectors have not gone without challenges with limitations such as limited infrastructure development; poverty and inequality; unemployment of over 20 percent; disease, which has dampened productivity; and lack of proper governance and accountability, which has created a habitat for an increase in cases of corruption in state and private entities. The findings of the study with the lessons learned from other studies with similar findings recommend that the government of Botswana should formulate suitable policies and strategies for services diversification. This is by expanding the market for the sector in areas such as tourism that were impacted by the COVID-19 pandemic, escalating investments by instituting strategies to attract and grow domestic and foreign investments, and improve on management of institutions and resources.

## 1. Introduction

Services are an important aspect of every country’s economic development [1,2,3,4]. A well and efficiently set up services system is key to how an economy of a country is most likely to perform as it promotes and majorly influences other sectors such as the agriculture sector as well as other secondary sectors [4,5,6,7]. In the absence of services such as insurance, transport, finance, tourism, marketing, or telecommunications, it would be difficult or almost impossible for other sectors to efficiently operate or even be competitive in the international market [5,8,9,10]. It is worth noting that the service sector also contributes to job creation in a country, as was the case in most developing countries where structural changes in terms of employment were witnessed between 1998 and 2004 [11,12]. Before then, the agricultural and construction sectors provided the most employment opportunities, but with the emergence of the service sector after 1991, this changed as it created more employment opportunities than any other sector in the country [11,12,13]. On a global scale, the value of the services added and contribution share to GDP has sharply increased in almost all the countries over the years [14]. In 2015, services contributed to about 69 percent of GDP from 63 percent recorded in 1997, with it specifically contributing about 74 percent of GDP in high-income countries which was an improvement on the 69 percent recorded in 1997 [15,16]. However, the increase was more noticeable in low- and middle-income economies where it moved from 48 percent to 57 percent during the same period, representing about a 9 percent increase [16]. As the services share of GDP increased, the other sector’s contributions declined [16]. For instance, statistics showed that within the same period, 1997 to 2015, the industrial sector’s contribution to GDP, high-income countries, and low- and middle-income countries had declined from 31.2 percent to 27.3 percent, 28.4 percent to 24.7 percent, and 37.7 percent to 33.6 percentage, respectively [15,16]. In developing worlds such as the SSA countries, the services market is relatively small compared to other developed markets as it accounts for only about 1.8 percent of global value-added as of 2014 [17]. However, the sector’s output in the region has experienced rapid growth in recent years, with a compound annual growth rate (CAGR) of 6.3 percent during the period 2004 to 2015, surpassing the world services growth rate of 2.6 percent by more than the double margin [17]. In 2015, the services sector contributed about 58 percent of GDP in SSA, which was a significant increase from the 47.6 percent observed in 2005 [16]. Despite this being the case, it has been observed that on average, the contribution of the services to SSA’S GDP has been lower than the global average in 2014 which stood at 68.5 percent [16]. Individually, each country’s services sector’s contribution to its GDP differs; for example, in countries such as Cape Verde and Mauritius, the services sector contributed over 70 percent of its GDP in 2015, with services such as finance and business having a much larger contribution. On the other hand, this was not the case in countries such as Chad and Sierra Leone where the services sector’s share of GDP was only about 33.4 percent and 33.9 percentage, respectively, during the same period [14,17]. According to the latest available data, Nigeria and South Africa are two of SSA’S largest economies which account for the most significant share of GDP in the region, with statistics showing a share contribution of 27.8 percent and 29.9 percent respectively [16,18]. These are followed by Sudan which is considered the 3rd largest contributor to SSA’s GDP, with a services value-added share of 3.6 percent. Other notable countries with at least a 2-percentage contribution include Kenya, Ghana, Ethiopia, and Tanzania [16]. The contribution of services to Botswana’s GDP was substantial and more than that of most SSA countries, with the contribution as a percentage of GDP in 2019 being pegged at 60.72 percent [16]. Rostow in his modernization theory argued that for a country to develop, it must go through five stages of development which include traditional, preconditions for take-off, take-off, drive to maturity, and high mass consumption [19,20]. Industrialization in Rostow’s vein has been seen as one of the key drivers in a country’s development [19,20]. In recent years, as countries have undergone development, there has been an emergence of the service sector which has been growing at a rapid rate in developing countries. The productivity per worker in the services sector is now higher than that of the agriculture and industrial sector, with the former being stagnant in most parts of the developing world, new services have emerged, further adding to an already existing service sector making the economy to grow [21,22]. Similarly, in one of the structural transformation theories, Ranis and Lewis agreed that as a country undergoes development, factors of production are reallocated from an agricultural sector to an industrial sector [16,23]. Eventually transformed from a heavy emphasis on the traditional agriculture economy to a more service and industrial one [8,9,10,24,25]. The contribution to GDP by the industry sector eventually surpasses that of the agricultural sector [19]. Additionally, the service sector has contributed towards income generation (for example through tourism) which is essential for economic development [8,9,10,24,25,26,27]. Furthermore, over the years, it has proven to be a key contributor to employment in developing countries, particularly for middle- and lower-income countries. The service sector also aids in the functioning of other sectors, hence improving it could be key to developing other sectors, hence ensuring sustainable economic growth. Botswana, a Sub-Saharan country, was not seen to have any exception growth when it got its independence in 1966. However, through industrialization, it is now known to have one of the strongest and fastest growing economies in Africa, with an average GDP growth rate of about 4 percentage per annum, which is expected to reach 4.7 percentage by 2022 according to the International Monetary Fund (IMF) recent report [28]. Botswana’s main GDP share contributors include the agriculture, industry, mining, and services sector [16]. The economy remains heavily dependent on diamond mining which has made it highly vulnerable to external shocks arising from prices fluctuations, hence emphasizing on the fundamental importance of other income generating sectors such as the service sector is essential. Over the years, there has been a rapid increase in the services value added to GDP with recently acquired data showing that the sector had an increased share of 55 percentage in 2010 to about 65.76 percent in 2020 surpassing the value-added share of the other two sectors [16]. Despite these positive figures, the growth has been too low to be able to attain Botswana’s development objectives as well as be able to create employment, hence the government is still looking at further expanding the industry sector through the introduction of ‘hubs’ in the health, education, innovation, financial services, and tourism sectors. Furthermore, these objectives can be met by building more services sector institutions such as banks and health facilities as well as setting up strict policies which protect the said sector. In seeking to determine how services impact Botswana’s GDP, this paper, seeks to focus on the relationship between and the impact of services on GDP, determining whether services contribute to GDP and to what extent, how services affect other sectors, and finally making policy recommendations on how Botswana can apply these policies in order to improve its services sector to be less reliant on diamond mining which is more vulnerable to prices volatilities. This article tests against the hypotheses of Rowstow’s modernization theory [19,20], and Ranis and Lewis structural transformation theory [23], that a nation’s GDP growth increases culminating from the growth in its services sector. This will be necessitated by answering the research question, “how and to what extent have services impacted the GDP, and economic growth in Botswana?”. The novelty of this paper is being the first to quantify the impact of services on Botswana’s GDP at a country level as well as showing the contribution of other sectors to GDP of Botswana. Similar studies conducted by various researchers in different parts of the world argued and concluded that there exists an impact culminating from services to GDP growth [8,9,10,11,12,22,24,25,29,30,31,32,33,34,35,36,37,38,39,40]. Other studies further acknowledged that specialized services in energy [39], finance development [4,7,9,10,33], healthcare [29,33,35], technology [8], and investment and service exports [24,25,40]. These conclusions are affirmed by previous studies supported by the modernization and structural transformation theories are the basis of a hypothesis that this paper seeks to test. The rest of this paper is arranged as follows. Section 2, which follows, gives an overview of the economy and services, which also pays attention to the macroeconomic indicators, the contribution of each sector to GDP, the challenges faced, and some current policies. Section 3 considers the data and methodology of the empirical measurement used in quantifying the effect of the services on GDP. Section 4 presents the empirical results and discussion, with a focus on empirical examples which the country can derive lessons from. Finally, Section 5 concludes and makes policy recommendations on how the services sectors can further enhance economic sustainability for Botswana serving as a model for other SSA economies.

## 2. Overview of Services and Botswana’s Economy

This section looks at the recent macroeconomics and the distribution of activities towards GDP, as well as policies and challenges affecting services and development in Botswana.

### 2.1. Macroeconomic Indicators and Distribution of Services Activities towards GDP

Table 1 below shows the performance of the macroeconomic indicators GDP per capita, GDP growth, inflation rate, and unemployment rate for the period from 2016 to 2020, with GDP per capita indicated in constant 2015 USD.

**Figure 1:**
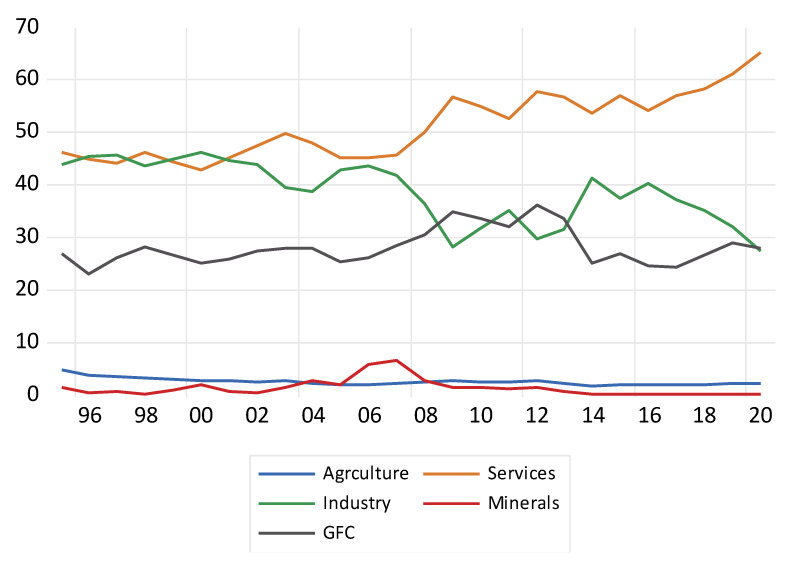
Sectors’ contribution to GDP in percentages, 1995 to 2020. Note: Agriculture is a proxy for the indicator agriculture, forestry, and fisheries. This will be used similarly on several figures and tables. Source: Authors’ computations from World Bank database [16].

As noted in the above Table 1, Botswana’s GDP per capita was 6729.066, 6855.16, 6973.099, 7027.045, and 6299.209 USD for the years 2016, 2017, 2018, 2019, and 2020, respectively. In the same focus period, the respective GDP growth rates were 7.03, 4.00, 3.98, 2.99, and −8.49 percent. The economy in the year 2020 was negative as the effect of the COVID-19 pandemic on the economic growth of Botswana became eminent as many businesses were slowed down. In the years 2016, 2017, 2018, 2019, and 2020, the rates of inflation rates were 2.814, 3.308, 3.238, 2.772, and 1.890 percent, respectively. For the same focus period, the respective unemployment rates were 21.029, 21.566, 22.071, 22.61, and 24.93 percent. Figure 1 below illustrates how the following sectors agriculture, forestry, and fisheries; services; industry; gross fixed capital (GFC); and mineral rent have contributed to Botswana’s economy as a percentage of GDP over the period 1995 to 2020. Over that period, services contributed the most to Botswana’s GDP, averaging between 45 and 55 percent contribution. This was seconded by industry, which contributed an average of around 40 percent during the same period. During the same period, GFC averaged around 20 percent. Agriculture, forestry, and fisheries as well as mineral rent had the least contributions to GDP of less than 10 percent. Figure 1 below shows the graphical representations of the contribution of agriculture, industry, services, mineral rent, and gross fixed capital as percentages of GDP.

**Figure 2:**
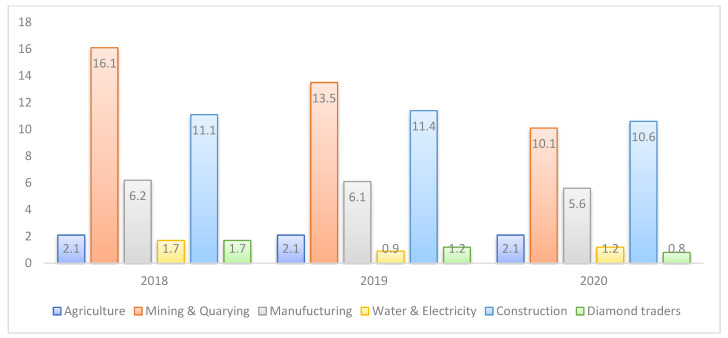
Distribution of GDP by Economic Activity (percentage of GDP). Source: Statistics Botswana [41].

Figure 2 below shows the distribution of GDP by economic activity for the years 2018, 2019, and 2020. Water and electricity contributed to GDP with 1.7, 0.9, and 12 percent in the years 2018, 2019, and 2020, respectively. In the same respective period, agriculture contributed 2.1 percent across all the years. For the periods 2018, 2019, and 2020, manufacturing contributed 6.2, 6.1, and 5.6 percent, while in the same respective period construction contributed 11.1, 11.6, and 10.6 percentages. Diamond traders contributed 1.7, 1.2, and 0.8, respectively. Moreover, mining and quarrying contributed 16.1, 13.5, and 10.1 in the years 2018, 2019, and 2020, respectively. During that period, services had the highest contribution to GDP, contributing 58.2, 60.72, and 65.76 percent in the years 2018, 2019, and 2020, respectively. The contribution of services was pronounced with many institutions offering services in 2020, with the country having over 12 banks, over 23 insurance companies, over 50 tertiary institutions, nearly 650 health institutions with over 26 public and the rest privately owned, over 20 transportation and logistics companies, and 3 telecommunications companies servicing a population of 2 million plus people [41].

The next sub-section looks at the policies related to and challenges that impacted the provision of services in the country.

### 2.2. Policies on and Challenges Affecting Services and Development in Botswana

To improve the service sector in Botswana, the government set up its primary focus on factors that change the growth of the services sector. Such factors include gross national expenditure, domestic credit to the private sector, and GFC formation [42]. It has also set up a policy that increases spending on the service sector and its sub-sectors. Additionally, the banking sector offered credit to the private sector to improve the growth of the sector, through a well-functioning and developed financial system. Other policies included the introduction of ‘hubs’ in the health, education, innovation, financial services, and tourism sectors [43,44]. However, Botswana continued having implementation challenges, some of which included the persistence of the human immunodeficiency virus/acquired immunodeficiency syndrome (HIV/AIDS) with a high prevalence rate of at least one in every five tested citizens according to the UNICEF report for 2022 [45]. Moreover, the country was impacted by slow economic diversification, rising youth unemployment, poverty, and social inequality [46]. This has affected the government’s efforts to achieve its aims. HIV/AIDS has been one of the country’s main challenges. Despite Botswana only recording its first case in 1985, it has become one of the worst hit countries being the fourth most hit country globally just after South Africa, Eswatini, and Lesotho. This was despite all efforts made to contain the disease such as the designation of a special coordinating institution, the National AIDS Coordinating Agency (NACA), assistance from international organizations such as the African HIV/AIDS Partnerships (ACHAP), and the US Government funded Presidential Emergency Plan for AIDS Relief (PEPFAR), which reduced the productiveness of its population. Another major issue has been that of slow diversification of the economy [46].

Botswana has for a long time depended on diamond mining, and its economy was at one point amongst the highest in the world for two decades. However, the dependence caused concern for the stakeholders in the economy as diamonds accounted for over 40 percent of its exports which became a major challenge during the global economic downturn, hence leading to a decline in mineral revenues which has resulted in a lack of benefits derived from the industry [44]. Lack of infrastructure development has also been an area of major concern in policy implementation; most projects are delivered very late, this coupled with poor workmanship and budget overruns has largely contributed to the challenges facing Botswana’s services sector and development. An example is that of the three sports stadiums which were supposed to be due for completion before the 2010 soccer world cup but were instead still incomplete by 2013. Another is the Sir Seretse Khama Airport in Gaborone, which despite having already gone beyond its initial budget is still incomplete. Corruption, as well as the industry, tends to defeat the idea of citizen involvement in the economy and continues to pose another major challenge in Botswana. These challenges have all been largely due to various reasons which include declining public accountability, lack of commitment to reforming the public sector, and a decline in the commitment by state authorities [42,46]. The next section is the data and methods.

## 3. Methodology

The empirical analysis applied the econometrics procedure in examining the impact of services on GDP in the economy of Botswana. The data used in the study were collected from the World Bank’s world development indicators’ annual database with a specific focus on the period 1975 to 2020 [16]. Analysis and economic procedures were carried out with the EVIEWS 12 software, econometrics forecasting software (created by Quantitative Micro Software, and currently owned by IHS Markit, London, UK). The variables of interest are indicated in Table 2.

All variables (except for mineral rent) were converted to logs and interpreted as elasticities, making them easy to interpret and limiting the threat of heteroskedasticity. The structural transformation [23] and modernization [19,20] theories justify the inclusion of all the variables to the model, thanks to their role in services serving as a catalyst to stimulate economic growth. Additionally, GFC contributed immensely, well over 30 percent to Botswana’s GDP over the focus period [16], while mining has over the years served as the country’s main economic activity [41]. These reasons warrant the inclusion of the variables described in Table 2 to the model. The empirical and econometric steps follow.

### 3.1. Empirical and Econometric Steps

The model’s general formula is as follows:

GDP = f (Agriculture, Services, Industry, Minerals, GFC)(1)

The model is represented in its stochastic form as:

GDP = a_0_ + a_1_Agriculture + a_2_Services+ a_3_Industry + a_4_Minerals +a_5_GFC + U_t_ (2)

where a_0_ is intercept; while a_1_, a_2_, a_3_, a_4_, and a_5_ represent coefficients for the variables shown in the general formula and U_t_ is the unobserved stochastic variable (term). The formula and the econometric procedures carried out in this paper are like those of many other researchers who used the same variables to conduct the analysis [22,38].

#### 3.1.1. Unit Root Test

An important step before carrying out any econometric procedure is a unit root test, which indicates whether the variables in the sample data are stationary or not to avoid running a spurious regression if any of the variables of interest were nonstationary which makes it hard and unreliable for any inference when carrying out estimations [47,48,49]. In trying to find out if there is any presence of stationarity in the variables of interest, the two most common test procedures used are the and the Phillips–Peron (PP) [50] and the augmented Dickey–Fuller (ADF) [51] test. However, the most preferred between the two is the Augmented Dickey–Fuller (ADF) as it takes care of any problems of serial autocorrelation [51]. A test for the impact of structural breaks conducted on the variables of interest was necessary and also saved as a confirmatory test to the ADF and PP test, using the Zivot–Andrews (Z-A), which was superior to other tests such as the PP and ADF tests which are not able to account for any shock and structural breaks in the model, by recording them as a unit-root, a premise that the Z-A test can account for [52]. Several scholars have compared time series, pertaining to the unit root test, using a combination of alternatives, such as the ADF, PP, and Z-A, to reach a proper compromise or consensus on the most appropriate econometric procedure [53,54,55,56,57,58], and in the case of this article the ARDL Bounds test.

Concerning the ADF, it takes the general form as shown:

(3) $$\Delta Y_{t}\;=\;\beta_{1}\;+\;\beta_{2}\;+\;\delta \;Y_{t-1}\sum_{i=1}^{m}\alpha \Delta Y_{t-1}\;+\;E_{t}$$

where:

$\Delta Y_{t}$ = related variable; $\beta_{1}$, $\beta_{2}$, δ,α= parameters in the model; *t* = time trend; and $E_{t}$ = Gaussians white noise with its mean = 0; and possible autocorrelation which is represented by time *t*.

#### 3.1.2. ARDL Bounds Test

After carrying out the unit root test and finding out the order of integration of the variables, the ARDL Bounds test is used as it is capable of running estimation for time series on variables which has the order of integration such as I(0) and I(1) or even a mixture of both but strictly not of a higher order I(2) [49]. This procedure goes beyond the limitations of Engle and Granger [47], and Johansen and Jeselius [53], which constrains the cointegration steps only to variables with the same order of integration as the ARDL The Bounds test can run a regression with variables of order I(0), I(1), or a combination of both and hence making it superior, a proposition that has been supported by several scholars pertaining to models with similar time series properties [49,54,55,56,57,58,59]. The optimal lag determination criteria adopted for each of the variables were computed automatically using the Akaike Information Criterion (AIC) [60,61], because it can suit the small sample sizes and also reduce any chances of underestimating the lags in the sample as it improves chances of determining the correct lag length unlike the other methods such as the Sequential modified LR test statistic, Final prediction error, Schwarz information criterion, and Hannan–Quinn information criterion [60,61].

The ARDL model is represented as follows:

(4) $$\begin{array}{c} \Delta GDP=\;\alpha_{0}+\sum_{i=1}^{p}\alpha_{1i}\Delta {GDP}_{t-p}\;+\;\sum_{i=1}^{p}\alpha \Delta_{2i}{Agriculture}_{t-p}\;+\;\sum_{i=1}^{p}\alpha_{3i}\;\Delta \mathrm{Services}\\ t-p+\sum_{i=1}^{p}\alpha_{4i}\Delta {Industry}_{t-p}+\sum_{i=1}^{p}\alpha_{5i}\Delta {\mathrm{Minerals}}_{t-p}+\sum_{i=1}^{p}\alpha_{6i}\;\Delta {\mathrm{GFC}}_{t-p}+\\ \lambda_{1}{\mathrm{GDP}}_{t-1}+\lambda_{2}{\mathrm{Agriculture}}_{t-p}+\lambda_{3}\;{\mathrm{Services}}_{t-p}+\\ \lambda_{4}{\mathrm{Industry}}_{t-p}+\lambda_{5}\;{Minerals}_{t-p}+\lambda_{6}\;{GFC}_{t-p}+E_{t} \end{array}$$

Δ = the difference operator; *p* denotes optimal lag length; while $\alpha_{0}$ is the constant term; $\alpha_{1i}\;,\;\alpha_{2i}\;,\;\alpha_{3i}\;,\;\alpha_{4i},\;\alpha_{5i},\;\alpha_{6i}$ are conficients of variables; Δ are error correction dynamics; $\lambda_{1}$, $\lambda_{2}$, $\lambda_{3}$, $\lambda_{4}$, $\lambda_{5}$, $\lambda_{6}$ are long-term coefficients, $E_{t}$ = White noise disturbance term.

The procedure for the cointegration test for ARDL Bounds test follows the F-test with a decision to reject the null hypothesis that cointegration is not present if the F-statistic is higher than the upper critical value or above the upper bound I(1) and lower bound I(0) and fail to reject the null hypothesis if the F-statistic is smaller than the lower critical value or below the lower bound I(0), with the cointegration results inconclusive if the cointegration test entails that the F-statistic value lies between I(0) and I(1) bounds [38,39].

#### 3.1.3. Diagnostic Tests

**Figure 3:**
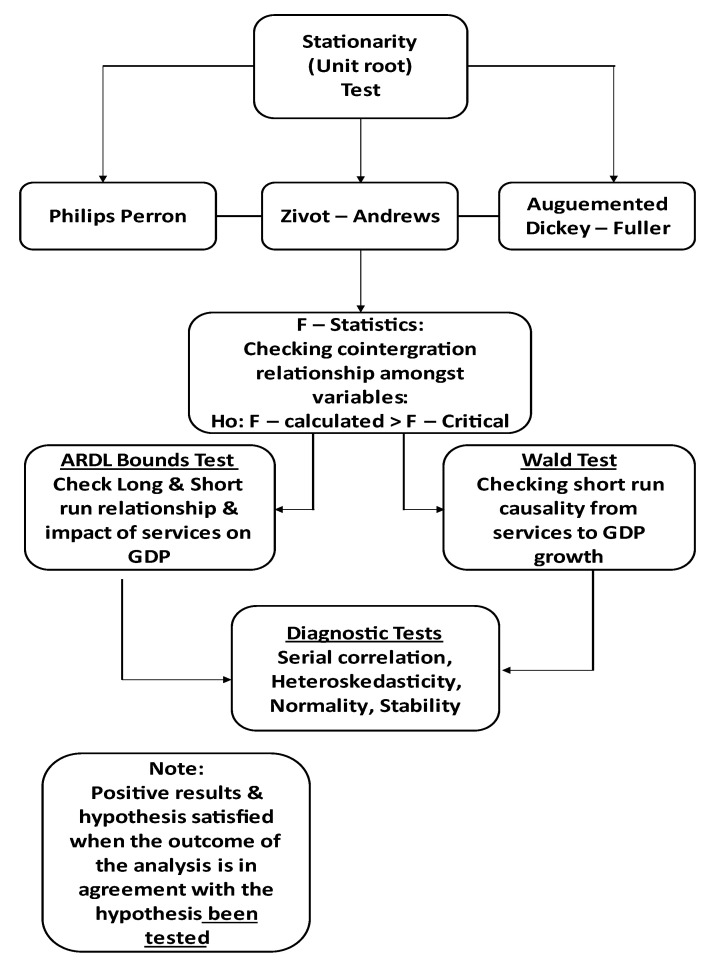
Theory and methodological flow chart.

The post-estimation tests carried out in the study include the test for heteroscedasticity, autocorrelation, model stability, and normality. The preferred null hypothesis is no autocorrelation, heteroskedasticity, and the presence of normality [49,54,55,56,57,58,59,61], with the acceptance having a lower F-statistic and a higher corresponding probability value, greater than 5 percent. The Cusum and Cusum of squares tests for model stability and stability without the effect of breaks, respectively, were also conducted [61]. The flow chart summarizing the empirical procedure used in answering responding to the paper’s objectives, hypothesis, and questions is indicated in Figure 3, after which the results and discussion will follow in the subsequent section.

## 4. Results and Discussion

Table 3 below shows the descriptive statistics of the variables of interest GDP; agriculture, forestry, and fisheries; services; industry; mineral rent; and gross fixed capital.

As noted in Table 3 above, the maximum, minimum, mean, and standard deviation for GDP were 16,188,225,469, 932,963,477.5, 7,302,798,762, and 4,696,841,763 USD, respectively. The similar respective values for agriculture, forestry, and fisheries were 315,250,945.49, 124,022,755.9, 218,365,593.6, and 51,290,552 USD, while those of services were 9,635,264,765, 298,412,627.3, 3,505,455,300, and 2,921,953,703 USD, respectively. Concerning industry, the values were 5,862,333,591, 481,292,062.3, 3,695,669,670, and 1,693,761,635 USD for the maximum, minimum, mean, and standard deviation, respectively, while the similar respective indicators for gross fixed capital were 5,650,269,924, 219,525,876.3, 1,741,000,002, and 1,441,768,688, USD. The maximum, minimum, mean, and standard deviation for minerals were 6.493806, 0.006359, 1.520492, and 1.738143 percent, respectively. Table 4 below indicates the results of the stationary tests for the variables of interest GDP; agriculture, forestry, and fisheries; services; industry; mineral rent; and gross fixed capital. Table 4 shows the unit root test for the variables of interest.

As noted in Table 4 above, the GDP unit root results exhibited an I(1) level of integration, with the ADF, PP, and the Z-A having higher and statistically significant t-statistics absolute values of 5.808603, 5.729063, and 6.365175, respectively, which were higher than the respective t-critical absolute values of 4.180911, 3.515523, and 4.859812, with the Z-A test having 1988 as the break year. Agriculture, forestry, and fisheries had the level of integration I(1), with statistically significant and higher absolute t-statistic values of 7.357599 and 7.324190 using the ADF and PP, respectively, against its respective t-critical absolute values of 3.515523, and 3.515523. Agriculture, forestry, and fisheries also had a level of integration I(0) for the Z-A test with a statically significant *p*-value, with a higher absolute t-statistic of 7.090117, against the t-critical value of 4.859813, with 2001 as its break year. The services unit root results exhibited an I(1) level of integration, with the ADF, PP, and the Z-A having higher t-statistics absolute values of 4.330314, 4.366795, and 5.437097, respectively, against its respective t-critical absolute values of 3.515523, 3.515523, and 4.859812, with significant *p*-values, with Z-A test having 1983 as the break year. The industry unit root results exhibited an I(1) level of integration, with the ADF, PP, and the Z-A having higher t-statistics absolute values of 4.330314, 4.366795, and 5.437097, respectively, against its respective t-critical absolute values of 3.515523, 3.515523, and 4.859812, with significant *p*-values, with Z-A test having 1983 as the break year. Minerals had the level of integration I(0), with significant and higher absolute t-statistic values of 3.707501 and 3.807798 using the ADF and PP, respectively, against their respective t-critical absolute values of 3.513075 and 3.513075. Minerals also had a level of integration I(1) for the Z-A test with a statically significant *p*-value, with a higher absolute t-statistic of 7.560210, against its respective t-critical absolute values of 4.859812, with 1983 as its break year. Gross fixed capital was integrated of order I(0) using the ADF test, with a statistically significant t-statistic, having a higher absolute t-statistic of 3.826440 against its respective t-critical absolute values of 3.513075. In like manner, the Z-A test was integrated of order I(0), and was statistically significant, having a higher absolute t-statistic of 7.128090, against its respective t-critical absolute values of 4.859812, and a break year of 1987. Concerning the GFC unit root, using the PP test, the variable was integrated of order I(1), and statistically significant t-statistic, with a higher absolute value of 14.98628, against its respective t-critical absolute values of 3.515523. These results with a combination of ADF, PP, and Z-A for the variables have shown a mix of results, a combination of I(0) and I(1) levels of integration, making the ARDL Bounds test the appropriate procedure as suggested on the next section. Table 5 shows the cointegration and ARDL Bounds test results procedure. Concerning the ARDL Bounds tests, in Table 5 below, the AIC criterion selected the model’s optimal lags of 2, 0, 2, 2, 1, 1 for the variables GDP; agriculture, forestry, and fisheries; services; industry; mineral; and GFC, respectively (for the original ARDL estimation output, see Appendix A). As noted in the cointegration and ARDL output in the Table 5 below, the variable GDP, services, industry, minerals, and gross fixed capital converged to equilibrium at the speed of 46.89 percent, as indicated by the cointegration coefficient of 0.468902 (in absolute value). Intuitively, this means that the above variables to 2.13 years converge to equilibrium and impact GDP over the long run. The cointegration output was supported by the F-statistics, which was higher than the respective critical values at both the higher I(1) and lower bounds I(0). The F-statistic 8.416563 was higher than the I(1) bounds 3.79, 4.25, 4.67, and 5.23 at 10, 5, 2.5, and 1 percent, respectively. Moreover, it was higher than the I(0) bounds 2.75, 3.12, 3.49, and 3.93 at 10, 5, 2.5, and 1 percent, respectively. The model indicated that 97.7 of the variations in GDP were explained by the explanatory variables in the model observed by an r-squared of 0.977. The model was well fitted with a statistically significant F-statistic, which had an F-statistic value of 165.4724. The coefficients of the ARDL model in Table 5 were elasticities interpreted as ordinary least squares. As indicated in Table 4, a percent increase in services lead to GDP growth by 0.328 percent, and this coefficient was statistically significant with a significant *p*-value of less than 5 percent. The respective percentage in other variables industry, minerals, and gross fixed capital impacted GDP growth by 0.446, 0.0008, and −0.02 percent, respectively.

Table 6 below indicates the long-run impacts on GDP from the variables agriculture, forestry, and fisheries; services; industry; minerals, and gross fixed capital.

As illustrated in Table 6 above, over the long run, the impact of agriculture, forestry, fisheries; services; and industry was statistically significant, with a percentage change in these variables positively impacting GDP by 0.201, 0.241, and 0.452, percent, respectively. The impact of minerals and gross fixed capital on GDP was not statistically significant at 5 percent, with their respective percentage change impacting the change in GDP by −0.006 and 0.0463 percent, respectively, with mineral rent having a negative coefficient. Table 7 shows the causal impact used to predict changes in GDP culminating from changes in services, using the Wald Test.

**Figure 4:**
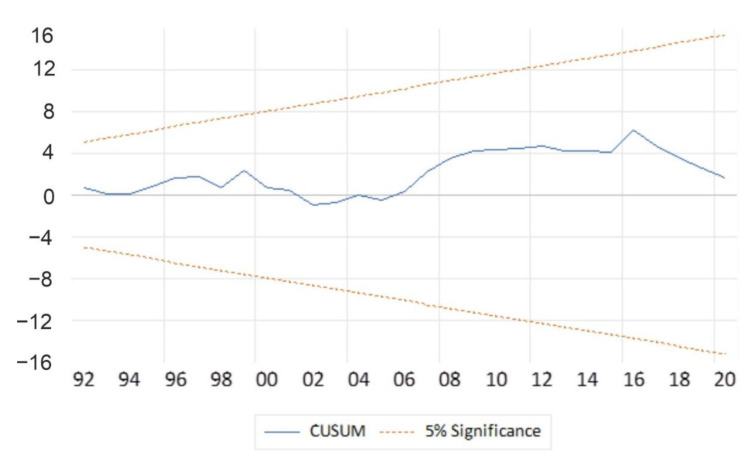
Stability tests (Cusum test). Source: Authors’ computations (2022).

As indicated in Table 7, the F-statistic had a respective probability of less than 5 percent, implying that a change in services (causes) influences the change in GDP in the short run. The model was checked for the post-estimation (diagnostics) test as indicated in Table 8 and Figure 3 and Figure 4. The post-estimation tests in Table 8 were for autocorrelation, heteroskedasticity, and normality, with respective *p*-values of 0.4563, 0.6476, and 0.415271. The results were desirable and they implied the absence of autocorrelation, heteroskedasticity, and the presence of a normal distribution of the residues of GDP of the model with the respective tests having probabilities values of 0.4563, 0.6476, and 0.415271 percentages, with these results indicated in Table 8 that follows.

**Figure 5:**
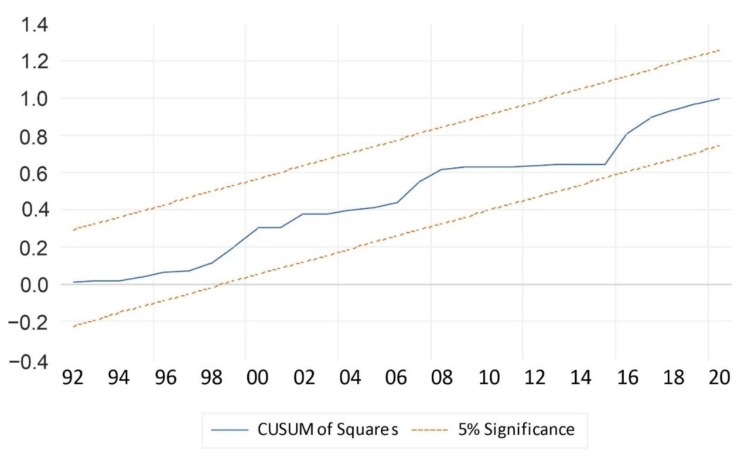
Stability test (Cusum squares test). Source: Authors’ computations (2022).

Figure 4 and Figure 5 show the stability test for the model using the Cusum test and Cusum squares test. The model in the figure below that the model is stable, as indicated by the red dotted lines rounding the blue line in between, which shows that the model and output line lies within the 10 percent boundaries and two standard deviations as illustrated in Figure 4. Figure 5 also has a similar intuition to Figure 4, except that it further confirms that the model was not impacted by structural breaks.

Table 7 and Figure 4 and Figure 5 all indicate that the model is well-fitted and reliable for interpretation, predicting output, and making reliable policy recommendations.

The services sector has been making huge contributions to the economy in terms of improvements in macroeconomic indicators such as national income, which has however been declining due to the recent persistence of the COVID-19 global pandemic. The success of the services sector has changed the economy in Botswana as it had been a source of economic growth and employment which has drastically improved the standards of living of people [15,16,28,41,42,43,44]. The sector’s growth has been of great benefit to the ever-growing large labor in Botswana through job creation. The demand for software accelerated investments in communications infrastructure-related industries such as IT-enabled services such as telecommunications supported other industries such as banking, insurance, hospitality, and aviation, which helped them grow. To achieve economic sustainability, a vital step was critically analyzing what other sectors in services sector contribute to the share of GDP including lowering the unemployment rates in Botswana as indicated in Table 1. As seen from the macroeconomic indicators for the period 2016–2020 in Table 1, Botswana’s GDP per capita increased steadily, reaching an all-time high in 2019 but sank by about 15.02% from 7027.045 recorded in 2019 to 6299.209 in 2020, which was due to declining demand and revenue from minerals, particularly diamonds which provided the economy with strong supplies of foreign exchange over the years. Similarly, the sluggish economic diversification and dependence on diamond coupled with the emergence of the COVID-19 pandemic exposed the already existing structural rigidities as well as made the economy vulnerable to external shocks and challenges that affected mineral-led economic growth which led Botswana’s economic growth to be declining over the same period and hit negative figures in 2020. Inflation has also been an issue of major concern in Botswana. However, due to lower demand and fuel prices, Botswana experienced prevailing low inflation rates from 2.8% in 2019 to 1.8% in 2020 which were way below the central bank’s target of between 3% to 6%. Another issue that has been an issue of concern in Botswana is the structurally high unemployment levels which were recorded at 21 percent in 2016 and rose to 24 percent in 2020 and was largely attributed to the lack of diversification. Over-dependence on mining robbed the economy of the chance to equip other sectors which would have largely helped in reducing unemployment. As earlier explained in Section 2, Figure 1 illustrated how agriculture, forestry, and fisheries, services, industry, GFC, mineral rent, and services have contributed to Botswana’s economy as a percentage of GDP during the period 1995 to 2020.

Over that period, services contributed the most to Botswana’s GDP, averaging between 45 and 55 percent over the period 1990 to 2020, with recent contributions to GDP of 58.2, 60.72, and 65.76 percentages in the years 2018, 2019, and 2020 respectively, as noted in Figure 2. This was followed by industry, which contributed an average of around 40 percent during the same period. During the same period, GFC average around 20 percent. Agriculture, forestry, and fisheries as well as mineral rent had the least contributions to GDP of less than 10 percent. Also in Figure 2, in the distribution of GDP by economic activity for the period 2018 to 2020, mining and quarrying remained the second major contributors to GDP, averaging between 10 and 16 percent, this was followed by construction at 10 to 11 percent while manufacturing, agriculture, diamond traders, and water and electricity had the lowest distribution share of below 10 percent. As mentioned earlier, Botswana has overly depended on mining, and hence the mining sector has the highest share distribution. Botswana’s government has made efforts to implement policies that ensure diversification and end reliance on mining. This could include redirecting investment towards other sectors in services such as tourism, banking, insurance, finance, and education among others. Policy challenges have however also been an issue that has largely contributed to the smaller distribution share of other sectors such as the agriculture and manufacturing sectors. Agriculture has also been vulnerable to climate change which reduced yields despite more than half of the population living in rural areas and being dependent on subsistence crops and livestock farming. Breaking it down further, water and electricity were recorded at 1.2 percent in 2020 which was an improvement on the 0.9 percent recorded in 2019. Even though the industry recorded the highest growth during the same period, its overall contribution to the economy remains the lowest. Year in and year out, growth in the agriculture sector has been weak when compared to the previous years, with the sector recording a contribution of 2.1 percent across 2018, 2019, and 2020. The stagnation in the real value added of the agriculture sector was mainly due to the decline in the livestock sub-industry. A decrease in the value added of mining and quarrying in 2020 was largely influenced by the decline in diamond and soda ash production value added. Diamonds were known to be luxury goods and as such, they were bound to fluctuate due to the appetite of reliable customers and external shocks which led to a significant drop in the global markets for diamonds, which (coupled with lower declarations of revenues to avoid paying royalties and taxes) then explains the decline in the contributions of diamond to GDP over the years.

In the short run and long run, the impact of services on GDP was positive and statistically significant at 5 percent with coefficients of 0.328 and 0.241, respectively, culminating from a one percent change on services towards the indicated percentage change in GDP as shown in Table 5 and Table 6, respectively, with Table 7 and the Wald test also showing causality culminating from services to GDP over the short run. The findings of this study have been endorsed by findings of related studies [8,9,10,11,12,22,24,25,30,31,32,33,34,35,36,37,38,39,40], which also agree with the hypothesis that services have a significant impact on economic growth and contributing to the country’s sustained economic development. Other related studies agreed with the notion that causality was inferred culminating from services to GDP [62,63,64,65]. Botswana, just like any other developing country in the SSA region is still in its initial stages of development. This paper showed that, in the long run, there is a significant impact of services on GDP growth, as were agriculture, industry, minerals, and GFC. This agrees with most studies that services are an essential part of the economy, and they could help to improve the standard of living of people as well as aid to boost economic growth if properly managed. Most countries where services have a significant impact on GDP have all put diversification towards sectors such as services as a priority, noting that it could link the economy to other industries such as agriculture, manufacturing, and mining [66,67,68,69], with the Botswana government acknowledging that the expansion of services sector could account for mitigating the effects on the environment, culminating from other activities such as mining and farming [70]. The improvements of Botswana’s economy culminated from the individual components have aided the country’s GDP growth. Sectors such as banking and finance, including insurance have aided economic growth through their contribution to job creation and finance investments. These findings and developments were noted in previous studies [4,7,9,10,33], and speak to the importance of this sectors in opening the door to economic prosperity, and the state is encouraged to ensure their favorable working environment. The government’s investments support is strongly appreciated and encouraged, including on the trade market where Botswana is a global exporter of beef, tourism, and diamond. The need to build on this comparative advantage is strongly encouraged, and other studies have shown that this is a noble cause beneficial for the state and region [24,25,40]. The country’s success and prioritization of the health sector by improving health and nutrition deserves recommendation, though challenges such as COVID-19 and HIV/AIDS in some areas were visible. The government’s success in fighting diseases speaks to a productive workforce, and Botswana having one of Africa’s highest per capita GDP, 7027.045 USD as at 2019 [16]. This proposition of a good health services supplementing GDP growth is a model to emulate and is related to realities of other examples [29,33,35]. Botswana can also play a key role in the provision of energy in southern Africa, thanks to government and other stakeholder support for education and technology. This is a worthwhile enablement to ensure economic sustainability as proposed by other studies [8,39,62]. The last section concludes by deriving lessons from our findings and making some policy recommendations on how best Botswana could use similar approaches to improve the services sector and GDP.

## 5. Conclusions and Recommendations

Efficient well-structured services and policy systems will aid the country in achieving economic sustainability. These policies are vital as they help boost the country’s economic welfare as well as provide employment which helps in eradicating the increasing unemployment rates and in return improves people’s standards of living. The government of Botswana has over the years made efforts to improve the services sector by increasing gross national expenditure on the sector as well as providing domestic credit facilities to people in the sector. Despite having a positive and significant impact on GDP, the services sector has experienced policy implementation challenges. Lack of infrastructure development has been a major concern in policy implementation. Poor workmanship and budget overruns have largely contributed to the challenges facing Botswana’s services sector. Considering the importance of services in economic diversification through the creation of employment, the main object of this paper was to analyze the impact of services on economic growth (GDP) which was found to be positive and significant. The novelty of the paper was to being the first in Botswana to investigate short-run and long-run dynamics between services and GDP in Botswana using a multivariate cointegration analysis to determine the long-run relationship between the variables GDP, agriculture, services, industry, minerals, and GFC. In analyzing this impact, the ARDL bound tests was applied for the period 1975 to 2020. As indicated in Table 5, a percent increase in services led to GDP growth by 0.328 percent, and this coefficient was statistically significant. In the long run, a percent change in services positively affects GDP growth by 0.241 and coefficients were statistically significant (Table 6). This paper through the modernization and structural transformation theory (which was proven true in the findings of this paper) has shown that Botswana’s services has contributed significantly to economic growth through activities supported through the financial sector and insurance, which has served as leeway for employment creation and financing innovation and investments. Education and health support have helped the country have a productive workforce with the state having one of the continents highest per capita GDP. The hope is to see Botswana capitalize on its international competitive advantage, with beef, tourism, and services exports helping to deescalate the over-reliance on mining, which was over the years impacted by price volatilities. As Botswana’s services sector lacks much diversification, this study with the lessons learned from other studies with similar findings recommends that the government of Botswana could apply the following four developmental strategies:

1. Formulate suitable policies and strategies for services diversification. The government can promote entrepreneurship in the services sector, by enhancing tax holiday for new local businesses and offer business opportunities to these businesses as a way of supporting the governments development agenda.
2. Provide and expand the market for the services sector, by enabling synergy in the cooperation amongst local business, foreign investments, and the state in enabling sustained economic development.
3. Increase investments in the services sector and its sub-sectors to make it competitive in the international market by prioritizing the services sector in the planning of national development agendas. This will be further enhanced by making informed decisions with an updated and significant result of research to avoid budget overruns as has been the case in the past.
4. Ensure that banks offer credit services to the private sector as this is vital for the growth of the services sector. This will help bridge the gap between innovation and accessibility to capital, making a pathway for the growth small and medium enterprises.

The sudden emergence of the COVID-19 pandemic presented the need for ensuring a sustainable economy through the services sector as an engine for growth through employment creation and serving as an aid to other industries. This research has shown that Botswana’s services sector still has great potential to develop even further, and that a well sustained services sector as catalyst for Botswana’s economic prosperity in view of the sustainable development goals, such as no poverty and decent economic growth among others. The main limitation of this study was that the quantifiable effects of COVID-19 on Botswana’s services and GDP were not captured in the econometric computations of the model due to the scantiness of the data. Future research could look at the effects of Botswana’s services sectors on the GDP growth in low-income and middle-income countries across the region and African continent.

## Figures and Tables

**Table 1:** Macroeconomic indicators 2016–2020.

Indicator	2016	2017	2018	2019	2020
GDP per capita	6729.066	6855.16	6973.099	7027.045	6299.209
GDP growth	7.036897	4.00345	3.980395	2.992738	−8.49289
Inflation	2.814958	3.308281	3.238016	2.772864	1.890359
Unemployment	21.029	21.566	22.071	22.61	24.93

Source: World Bank [16].

**Table 2:** Variables of interest.

Variable	Computation
GDP	real GDP constant 2015 USD (GDP is the dependant variable)
Agriculture	agriculture, fisheries, and forestry value-added constant 2015 USD
Services	services value added constant 2015 USD
Industry	industry value added constant 2015 USD
Minerals	mineral rent as a percentage of GDP
GFC	gross fixed capital constant 2015 USD

Source: World Bank database (2022) [16].

**Table 3:** Descriptive statistics USD.

Indicator	Maximum	Minimum	Mean	SDEV
GDP	16,188,225,469	932,963,477.5	7,302,798,762	4,696,841,763
Agriculture	315,250,945.49	124,022,755.9	218,365,593.6	51,290,552
Services	9,635,264,765	298,412,627.3	3,505,455,300	2,921,953,703
Industry	5,862,333,591	481,292,062.3	3,695,669,670	1,693,761,635
Minerals	6.493806	0.006359	1.520492	1.738143
GFC	5,650,269,924	219,525,876.3	1,741,000,002	1,441,768,688

Note: SDEV is the abbreviation for the standard deviation. All variables were measured in USD, except for Mineral, which is indicated as a percentage of GDP. Source: Authors’ computations from World Bank database (2022).

**Table 4:** Unit root tests.

Variable	Test	Level		1st Difference	
		Statistic	5 Percentage Critical	Statistic	5 Percentage Critical
GDP	ADF	−1.252485	−3.513075	−5.808603 *	−4.180911
	PP	−1.226981	−3.513075	−5.729063 *	−3.515523
	Z-A	−3.609328 (1987)	−4.859812	−6.365175 * (1988)	−4.859812
Agriculture	ADF	−2.623163	−3.513075	−7.357599 *	−3.515523
	PP	−2.695761	−3.513075	−7.324190 *	−3.515523
	Z-A	−7.090117 * (2001)	−4.859812		
Services	ADF	−0.319593	−3.513075	−4.330314 *	−3.515523
	PP	−0.807426	−3.513075	−4.366795 *	−3.515523
	Z-A	−4.074293 (1984)	−4.859812	−5.437097 * (1983)	−4.859812
Industry	ADF	−1.958670	−3.513075	−6.074625 *	−3.515523
	PP	−2.287473	−3.513075	−6.051098 *	−3.515523
	Z-A	−4.173820 (2008)	−4.859812	−6.710782 * (1983)	−4.859812
Minerals	ADF	−3.707501 *	−3.513075		
	PP	−3.807798 *	−3.513075		
	Z-A	−4.345889 (2005)	−4.859812	−7.560210 * (1983)	−4.859812
GFC	ADF	−3.826440 *	−3.513075		
	PP Z-A	−3.337899 −7.128090 * (1987)	−3.513075 −4.859812	−14.98628 *	−3.515523

Note: ADF is tested with constant and trend. * Indicates less than 5 percentage levels of significance. The year of structural break is indicated in brackets for the Z-A test. Source: Authors’ computations (2022).

**Table 5:** ARDL error correction regression.

Selected Model: ARDL(2, 0, 2, 2, 1, 1)				
ECM Regression				
Case 5: Unrestricted Constant and Unrestricted Trend				
Variable	Coefficient	Std. Error	t-Statistic	Prob.
C	1.014051	0.132587	7.648166	0.0000
@TREND	0.006988	0.000887	7.876244	0.0000
D(LGDP(-1))	0.565966	0.105733	5.352756	0.0000
D(LSERVICES)	0.328671	0.029906	10.99010	0.0000
D(LSERVICES(-1))	−0.071553	0.044434	−1.610322	0.1182
D(LINDUSTRY)	0.446737	0.017618	25.35677	0.0000
D(LINDUSTRY(-1))	−0.193498	0.045426	−4.259687	0.0002
D(MINERALS)	0.000800	0.000930	0.860610	0.3965
D(LGFC)	−0.020789	0.007670	−2.710569	0.0112
CointEq(-1) *	−0.468902	0.060939	−7.694554	0.0000
R-squared	0.977679	Mean dependent var		0.060540
Adjusted R-squared	0.971771	S.D. dependent var		0.052749
S.E. of regression	0.008863	Akaike info criterion		−6.417245
Sum squared resid	0.002671	Schwarz criterion		−6.011748
Log likelihood	151.1794	Hannan–Quinn criter.		−6.266867
F-statistic	165.4724	Durbin–Watson stat		2.157751
Prob(F-statistic)	0.000000			
F-Bounds Test		Null Hypothesis: No levels relationship		
Test Statistic	Value	Signif.	I(0)	I(1)
F-statistic	8.416563	10 percent	2.75	3.79
K	5	5 percent	3.12	4.25
		2.5 percent	3.49	4.67
		1 percent	3.93	5.23
t-Bounds Test		Null Hypothesis: No levels of relationship		
Test Statistic	Value	Signif.	I(0)	I(1)
t-statistic	−7.694554	10 percent	−3.13	−4.21
		5 percent	−3.41	−4.52
		2.5 percent	−3.65	−4.79
		1 percent	−3.96	−5.13

Source: Authors’ computations (2022). * *p*-value incompatible with t-Bounds distribution.

**Table 6:** Long-run impact of variables on GDP.

Levels Equation				
Case 5: Unrestricted Constant and Unrestricted Trend				
Variable	Coefficient	Std. Error	T-Statistic	Prob.
LAGRICULTURE	0.201394	0.056219	3.582300	0.0012
LSERVICES	0.241398	0.042270	5.710910	0.0000
LINDUSTRY	0.452878	0.033206	13.63844	0.0000
MINERALS	−0.006309	0.003174	−1.987795	0.0563
LGFC	0.046366	0.022935	2.021632	0.0525

Source: Authors’ computations (2022).

**Table 7:** Wald test for causality impact of services on GDP.

Test Statistic	Value	Df	Probability
F-statistic	17.46436	(3, 29)	0.0000
Chi-square	52.39307	3	0.0000
Null Hypothesis: C(4) = C(5) = C(6) = 0			
Null Hypothesis Summary:			
Normalized Restriction (=0)		Value	Std. Err.
C(4)		0.328671	0.046819
C(5)		−0.287031	0.071967
C(6)		0.071553	0.053538
Restrictions are linear in coefficients.			

Note: For original ARDL output, see Appendix A. Source: Authors’ computations (2022).

**Table 8:** Diagnostics test.

Problem	Test	*p*-Value
**Autocorrelation**	Breusch–Godfrey LM	0.4563
**Heteroskedasticity**	Breusch–Pagan–Godfrey	0.6476
**Normality**	Histogram	0.415271

Source: Authors’ computations (2022).

## Data Availability

Not applicable.

## Appendix A. Original ARDL Estimation output

Dependent Variable: LGDP				
Method: ARDL				
Date: 04/12/22 Time: 18:30				
Sample (adjusted): 1977 2020				
Included observations: 44 after adjustments				
Maximum dependent lags: 2 (Automatic selection)				
Model selection method: Akaike info criterion (AIC)				
Dynamic regressors (2 lags, automatic): LAGRICULTURE LSERVICES				
LINDUSTRY MINERALS LGFC				
Fixed regressors: C @TREND				
Number of models evaluated: 486				
Selected Model: ARDL (2, 0, 2, 2, 1, 1)				
Variable	Coefficient	Std. Error	t-Statistic	Prob. *
LGDP(-1)	1.097064	0.125146	8.766264	0.0000
LGDP(-2)	−0.565966	0.143648	−3.939937	0.0005
LAGRICULTURE	0.094434	0.022081	4.276650	0.0002
LSERVICES	0.328671	0.046819	7.020085	0.0000
LSERVICES(-1)	−0.287031	0.071967	−3.988377	0.0004
LSERVICES(-2)	0.071553	0.053538	1.336480	0.1918
LINDUSTRY	0.446737	0.022394	19.94863	0.0000
LINDUSTRY(-1)	−0.427880	0.057969	−7.381196	0.0000
LINDUSTRY(-2)	0.193498	0.060590	3.193554	0.0034
MINERALS	0.000800	0.001521	0.526171	0.6028
MINERALS(-1)	−0.003759	0.001299	−2.892989	0.0072
LGFC	−0.020789	0.010388	−2.001218	0.0548
LGFC(-1)	0.042530	0.011362	3.743339	0.0008
C	1.014051	0.616821	1.643996	0.1110
@TREND	0.006988	0.001653	4.227474	0.0002
R-squared	0.999892	Mean dependent var		22.51518
Adjusted R-squared	0.999840	S.D. dependent var		0.757585
S.E. of regression	0.009596	Akaike info criterion		−6.189972
Sum squared resid	0.002671	Schwarz criterion		−5.581726
Log likelihood	151.1794	Hannan–Quinn criter.		−5.964405
F-statistic	19140.65	Durbin–Watson stat		2.157751
Prob(F-statistic)	0.000000			
selection.				
Source: Authors’ computations (2022). * Note: *p*-values and any subsequent tests do not account for model.				

## References

[1] Leeson P.F. (1979). The Lewis model and development theory. Manch. Sch..

[2] Lewis W.A. (1984). The state of development theory. Am. Econ. Rev..

[3] Tignor R.L. (2020). W. Arthur Lewis and the birth of development economics. W. Arthur Lewis and the Birth of Development Economics.

[4] Kabat L., Cibák L., Filip S. (2020). The remittance inflows in Visegrad countries: A source of economic growth, or migration policy misting?. Entrep. Sustain. Issues.

[5] Dinu A.-M. (2017). The importance of tourism and touristic services in GDP. Quaestus.

[6] Omidi M., Min Q., Omidi M. (2017). Combined Effect of Economic Variables on Fraud, a Survey of Developing Countries. Econ. Sociol..

[7] Le T.T.H., Le T.D., Tran T.D., Duong Q.N., Dao L.K.O., Do T.T.N. (2021). Banking sector depth and economic growth: Empirical evidence from Vietnam. J. Asian Financ. Econ. Bus..

[8] Zatonatska T., Rozhko O., Tkachenko N. (2018). Modern trends of impact on economic development of countries: E-commerce and R&D. Mark. Manag. Innov..

[9] Emara N., El Said A. (2021). Financial inclusion and economic growth: The role of governance in selected MENA countries. Int. Rev. Econ. Finance.

[10] Marcelin I., Egbendewe A.Y., Oloufade D.K., Sun W. (2022). Financial inclusion, bank ownership, and economy performance: Evidence from developing countries. Finance Res. Lett..

[11] Eichengreen B., Gupta P. (2011). The Service Sector as India’s Road to Economic Growth (No. w16757).

[12] Eichengreen B., Gupta P. (2013). The two waves of service-sector growth. Oxf. Econ. Pap..

[13] Jain D., Nair K., Jain V. (2015). Factors affecting GDP (manufacturing, services, industry): An Indian perspective. Annu. Res. J. SCMS Pune.

[14] The Sub-Saharan African Services Economy: Insights and Trends—Tralac Trade Law Centre [WWW Document]. https://www.tralac.org/news/article/12337-the-sub-saharan-african-services-economy-insights-and-trends.html.

[15] Buckley P., Majumdar R. (2018). The services powerhouse: Increasingly vital to world economic growth. Deloitte Insights.

[16] World Bank (2022). World Development Indicators. https://databank.worldbank.org/source/world-development-indicators.

[17] Powell J. (2017). Sub-Saharan African Services Economy: Insights and Trends.

[18] Oh E. (2017). Nigeria’s Services Economy: The Engine for Future Growth.

[19] Rostow W.W. (1960). The Stages of Economic Growth. A Non-Communist Manifesto.

[20] Lhomme J., Rostow W.W. (1961). The stages of economic growth, a non-communist manifesto. Rev. Économique.

[21] Sharma R., Hazra S., Chitkara S. (2007). Measurement of GDP of Services Sector in the New Series of National Accounts Statistics. Econ. Political Wkly..

[22] Malik M.H., Velan N. (2020). An analysis of IT software and service exports from India. Int. Trade Politi-Dev..

[23] Islam R., Reddy D.N., Sarap K. (2017). Structural Transformation and Alternative Pathways to the Lewis Turning Point. Rural Labour Mobility in Times of Structural Transformation: Dynamics and Perspectives from Asian Economies.

[24] Gabriele A. (2006). Exports of services, exports of goods, and economic growth in developing countries. J. Econ. Integr..

[25] Park S. (2007). Trade in Services and Economic Growth. J. East Asian Econ. Integr..

[26] Singh H. (2015). Composition & Contribution of Service Sector in India, Jagran Josh. https://www.jagranjosh.com/general-knowledge/composition-contribution-of-service-sector-in-india-1446187472-1.

[27] Tekola H., Gidey Y. (2019). Contributions of micro, small and medium enterprises (MSMEs) to income generation, employment and GDP: Case study Ethiopia. J. Sustain. Dev..

[28] International Monetary Fund (2022). Botswana Economic Outlook. https://www.imf.org/en/Countries/BWA.

[29] Suzuki T., Hotta J., Kuwabara T., Yamashina H., Ishikawa T., Tani Y., Ogasawara K. (2020). Possibility of introducing telemedicine services in Asian and African countries. Health Policy Technol..

[30] Esfahani H.S., Ramírez M.T. (2003). Institutions, infrastructure, and economic growth. J. Dev. Econ..

[31] Bacovic M., Andrijašević Ž.M., Cerovic Smolovic J. (2022). Structural changes and growth in Europe: Are knowledge-intensive services changing paradigm of expansion of services as a long-term growth-diminishing factor?. Ekon. Časopis.

[32] Kizyma T.O., Lobodina Z.M., Khamyha Y.Y. (2019). Fraud with Financial Resources of the State: Types and Effect on the Shadow Economy. Financ. Credit Act..

[33] Mulungu K., Ng’Ombe J.N. (2017). Sources of Economic Growth in Zambia, 1970–2013: A Growth Accounting Approach. Economies.

[34] Chiu Y.-B., Zhang W., Ding K. (2021). Does Globalization Influence Inbound Tourism? Evidence from a Dynamic Panel Threshold Analysis. J. Travel Res..

[35] Wang F., Wang J.-D. (2021). Investing preventive care and economic development in ageing societies: Empirical evidences from OECD countries. Health Econ. Rev..

[36] Kouassi E., Akinkugbe O., Kutlo N.O., Brou J.M.B. (2018). Health expenditure and growth dynamics in the SADC region: Evidence from non-stationary panel data with cross section dependence and unobserved heterogeneity. Int. J. Health Econ. Manag..

[37] Doytch N., Uctum M. (2011). Does the worldwide shift of FDI from manufacturing to services accelerate economic growth? A GMM estimation study. J. Int. Money Financ..

[38] Yue X.-G., Liao Y., Zheng S., Shao X., Gao J. (2021). The role of green innovation and tourism towards carbon neutrality in Thailand: Evidence from bootstrap ADRL approach. J. Environ. Manag..

[39] Garba I., Bellingham R. (2021). Energy poverty: Estimating the impact of solid cooking fuels on GDP per capita in developing countries—Case of sub-Saharan Africa. Energy.

[40] Sharma R., Kautish P. (2020). Linkages between Financial Development and Economic Growth in the Middle-Income Countries of South Asia: A Panel Data Investigation. Vision J. Bus. Perspect..

[41] Statistics Botswana (2022). Botswana’s Social Economic Statistics. https://www.statsbots.org.bw/statistics-by-sector.

[42] Kaboyakgosi G., Marata K.P. (2013). An analysis of Botswana’s implementation challenges. PULA Botsw. J. Afr. Stud..

[43] Mogomotsi P.K., Kolobe M., Raboloko R., Mmopelwa G. (2018). The economic contribution of tourism to local communities: The case of Khumaga and Moreomaoto villages, Botswana. PULA Botsw. J. Afr. Stud..

[44] Raboloko M. (2018). Determinants of Service Sector Growth in Botswana.

[45] UNICEF (2022). Botswana: Health and Nutrition. https://www.unicef.org/botswana/health-and-nutrition.

[46] Ghebremusse S. (2018). Good Governance and Development in Botswana - The Democracy Conundrum. Law Dev. Rev..

[47] Engle R.F., Granger C.W.J. (1987). Co-Integration and Error Correction: Representation, Estimation, and Testing. Econometrica.

[48] Nelson C.R., Plosser C.I. (1982). Trends and random walks in macroeconmic time series: Some evidence and implications. J. Monet. Econ..

[49] Pesaran M.H., Shin Y., Smith R.J. (2001). Bounds testing approaches to the analysis of level relationships. J. Appl. Econom..

[50] Phillips P.C., Perron P. (1988). Testing for a unit root in time series regression. Biometrika.

[51] Dickey D.A., Fuller W.A. (1981). Likelihood Ratio Statistics for Autoregressive Time Series with a Unit Root. Econometrica.

[52] Zivot E., Andrews D.W.K. (2002). Further evidence on the great crash, the oil-price shock, and the unit-root hypothesis. J. Bus. Econ. Stat..

[53] Johansen S., Juselius K. (1990). Maximum likelihood estimation and inference on cointegration—With applications to the demand for money. Oxf. Bull. Econ. Stat..

[54] Adebayo T.S., Odugbesan J.A. (2021). Modeling CO_2_ emissions in South Africa: Empirical evidence from ARDL based bounds and wavelet coherence techniques. Environ. Sci. Pollut. Res..

[55] Ahmad M., Khattak S.I., Khan S., Rahman Z.U. (2020). Do aggregate domestic consumption spending & technological innovation affect industrialization in South Africa? An application of linear & non-linear ARDL models. J. Appl. Econ..

[56] Ahmadi M. (2021). Social and Economic Impacts of Elimination of Energy Price Distortion in Indonesia. Ph.D. Thesis.

[57] Alola A.A., Onifade S.T. (2022). Energy innovations and pathway to carbon neutrality in Finland. Sustain. Energy Technol. Assess..

[58] Chin L.C., Hoang T.C., Novak P., Jurigova Z., Kozubikova L., Zlamalova J. (2017). Effects of US Economic Policy Uncertainty on Stock Price in Vietnam: An Ardl Bound Test Approach. Finance and Performance of Firms in Science, Education, and Practice.

[59] Phiri J., Malec K., Kapuka A., Maitah M., Appiah-Kubi S.N.K., Gebeltová Z., Bowa M., Maitah K. (2021). Impact of Agriculture and Energy on CO_2_ Emissions in Zambia. Energies.

[60] Liew V.K.-S. (2006). Which Lag Length Selection Criteria Should We Employ? SSRN Scholarly Paper No. ID 885505.

[61] Woodridge J.M. (2018). Introductory Econometrics a Modern Approach.

[62] Hassan K., Abdullah A. (2015). Effect of Oil Revenue and the Sudan Economy: Econometric Model for Services Sector GDP. Procedia-Soc. Behav. Sci..

[63] Uddin M.M.M. (2015). Causal relationship between agriculture, industry and services sector for GDP growth in Bangladesh: An econometric investigation. J. Poverty Investig. Dev..

[64] Yetiz F., Özden C. (2017). Analysis of causal relationship among gdp, agricultural, industrial and services sector growth in Turkey. Ömer Halisdemir Üniv. İktisadi İdari Bilim. Fak. Derg..

[65] Katircioglu S. (2004). Co-integration and causality between GDP, agriculture, industry and services growth in North Cyprus: Evidence from time series data. Rev. Soc. Econ. Bus. Stud..

[66] Gordon J., Gupta P. (2005). Understanding India’s services revolution. India’s and China’s Recent Experience with Reform and Growth.

[67] Guo X., Shi J., Ren D., Ren J., Liu Q. (2017). Correlations between air pollutant emission, logistic services, GDP, and urban population growth from vector autoregressive modeling: A case study of Beijing. Nat. Hazards.

[68] Todaro M.P., Smith S.C. (2015). Economic Development.

[69] Phiri J., Malec K., Majune S., Appiah-Kubi S., Gebeltová Z., Kotásková S., Maitah M., Maitah K., Naluwooza P. (2021). Durability of Zambia’s Agricultural Exports. Agriculture.

[70] (2016). Botswana Statistics Botswana Environmental Statistics 2016. https://unstats.un.org/unsd/environment/Compendia/Botswana%20Environment%20Statistics%20Revised%20Version,%202016.pdf.

